# An Endemic Plant of the Mediterranean Area: Phytochemical Characterization of Strawberry Tree (*Arbutus unedo* L.) Fruits Extracts at Different Ripening Stages

**DOI:** 10.3389/fnut.2022.915994

**Published:** 2022-06-17

**Authors:** Pierpaolo Scarano, Rosa Guida, Daniela Zuzolo, Maria Tartaglia, Antonello Prigioniero, Alessia Postiglione, Gabriella Pinto, Anna Illiano, Angela Amoresano, Rosario Schicchi, Anna Geraci, Rosaria Sciarrillo, Carmine Guarino

**Affiliations:** ^1^Department of Science and Technology, University of Sannio, Benevento, Italy; ^2^Department of Chemical Science, University of Naples Federico II, Naples, Italy; ^3^INBB—Consorzio Interuniversitario Istituto Nazionale di Biostrutture e Biosistemi, Rome, Italy; ^4^Department of Agricultural, Food and Forestry Sciences, University of Palermo, Palermo, Italy

**Keywords:** *Arbutus unedo* L., MRM mass spectrometry, total phenolic compounds, radical scavenging activities, ripening process

## Abstract

This work focused on the extraction, quantification, and characterization of bioactive compounds of *Arbutus unedo* L. fruits, comparing the results obtained from the different ripening states. Extractions were performed by different methods (such as maceration extraction and ultrasonic extraction) and food grade solvents (aqueous and hydroalcoholic solvents) in each of the all ripening states (four states considered, associated with four different colors, i.e., green, yellow, orange, and red). The presence of (poly)phenols was quantified and characterized, and scavenging activity was determined by the Folin–Ciocâlteu reagent and the DPPH method, respectively. The content of bioactive compounds was characterized by LC-MS/MS, such as multiple reaction monitoring (MRM) mass spectrometry. The results showed that ultrasound-assisted extraction (UAE) performed better than maceration extraction; ethanol–water mixture extracts showed a more positive effect than the use of aqueous extracts regarding the content of total phenolic compounds. Overall, the total phenolic compounds in the EtOH:H_2_O mixture at a ratio of 7:3 (v:v) were higher than that of the other solvents for both extraction methods. Some bioactive molecules were characterized for the first time in the extracts of *A. unedo*. The chemical profile of the strawberry tree extracts depended on the degree of fruit ripeness. The results suggest that *A. unedo* fruits may be of great interest for food and nutraceutical applications.

## Introduction

The strawberry tree (*Arbutus unedo* L.) is an evergreen shrub species, usually not exceeding the 4 m, belonging to the family of the Ericaceae. It grows wild in various areas of the Mediterranean: it is present in fact in western, central, and southern Europe, in north-eastern Africa (excluding Egypt and Libya) and in the Canary Islands and in western Asia (1).

In the recent years, the species of *A. unedo* has increased dramatically, being used in landscape subdivision, protection from abiotic (fire) and biotic (pests) factors (2, 3). It has recently aroused a lot of interest in the producers. This interest derives mainly from the economic potential of fruit exploitation, both through its processing (liqueurs and wines, jams, honey, snacks, and yogurt) (4, 5) and its fresh consumption.

The fruit ripening process includes a series of physiological modifications that make the fruit palatable for consumption. The alterations mainly concern the cell wall softening process, the change in color due to the conversion from chloroplast to chromoplast, the enrichment of the content in carbohydrates and of volatile and semi-volatile compounds that will give the peculiar aromatic bouquet to the ripe fruit (6). The *A. unedo* fruits are spherical and have a strong color alteration during the ripening. The ripening starts from the green, unripe fruit that the plant produces between spring and summer, shift to the yellow and orange fruit present between summer and fall. At the end of ripening, its ripe fruits (when ripened in full) are dark red and about 2 cm of diameter.

All the botanical parts of the plant, both leaves and fruits, as well as roots and barks, are used in traditional folk medicine to prepare extracts and infusions with antiseptic, diuretic, laxative, and depurative properties in general (7–9). Extracts of the roots, in particular, have been the subject of *in vitro* study in the treatment of urological and dermatological problems (10). Extracts of *A. unedo*, again, have been the subject of study in the treatment of various cardiovascular (11) diseases (*in vitro* studies) such as hypertension (*in vitro* study), thrombosis, and atherosclerosis (*in vitro* and *in vivo* studies) (12–15) of diabetes (*in vitro* and *in vivo* studies) (16) and in the treatment of inflammatory diseases (*in vivo* study) (17). These effects, which mainly concern the extracts obtained from leaves, depend on the presence in relatively high quantities of polyphenols: these molecules, as is well known, are natural antioxidants present in plants and are studied for their possible use in the prevention of diseases and in reacting with free radicals. Leaves, in particular, are rich in polyphenolic compounds such as tannins, molecules with important anti-inflammatory and antimicrobial properties (18). Strawberry tree fruits and leaves have been characterized to study their beneficial properties for health. The presence of antioxidants, vitamins, and minerals has been confirmed in these extracts. As for the flowers, they have been still less studied (19).

In this work, after performing an analysis of the present literature, we have aimed to increase the knowledge on the fruits of the strawberry tree at different maturation, exploring the antioxidant activity according to the Folin–Ciocâlteu method, determining its scavenging activity through the DPPH method (2,2-diphenyl-1-picrylhydrazyl), characterizing the extracts with the support of liquid chromatography (LC) coupled to tandem mass spectrometry using the multiple reaction monitoring (MRM) ion mode approach. Each step was carried out to highlight the important difference in the production of bioactive compounds that occur for each state of ripeness of the fruit and that could open up new studies in the biological field: in fact, studies of cytotoxicity of the extracts on cells (not the subject of this work) are in progress. This type of characterization could make an important contribution to developing new products based on the fruits of *A. unedo*, which have an excellent potential to fight some diseases (20). We have also focused on the evaluation of two techniques for the extraction of bioactive compounds of interest, basing the evaluations on the results obtained from the analyses listed above.

## Materials and Methods

### Chemicals and Reagents

All solvents and reagents were of high quality with a high grade of purity. Ethanol ≥ 99.9% ACS for analysis was obtained from VWR International (Milano, Italy). Hydrochloric acid 37% RPE for analysis, potassium bicarbonate, anhydrous sodium carbonate for analysis, and gallic acid ACS for analysis were obtained from Carlo Erba (Milano, Italy). Methanol for HPLC, n-hexane anhydrous at 95%, chloroform for chromatography, and 1-butanol ACS reagent ≥ 99.5 were acquired from Sigma-Aldrich Chemical Company (Milano, Italy). Folin–Ciocâlteu reagent and acetonitrile hypergrade for LC–MS were purchased from Merck Millipore GmbH (Milano, Italy). 2,2-Diphenyl-1-picrylhydrazyl (DPPH**^⋅^**) was obtained from Alfa Aesar (Thermo Fisher Scientific companies in Rodano, Milano, Italy). Malvidin-3-O-glucoside, naringin, catechin, quercetin, gallic acid, vanillic acid, caffeic acid, ferulic acid, and amino acid mixture were purchased from Merck (Milano, Italy).

Bidistilled water was utilized throughout the preparation of the solutions. The extracts obtained from the different fruits of *Arbutus unedo* L. were used without any purification, and all the analyzed solutions, where necessary, were prepared by diluting the stock solutions of the extracts in water or in a hydroalcoholic solution.

### Plant Materials: Recovery and Storage

The fruits of *Arbutus unedo* L. (strawberry tree) with four different ripening (determined according to the external color: green, yellow, orange, and red) (henceforth referred to as V, G, A, and R, respectively) were harvested from June to November 2020 in South of Italy (41^°^0′28″80 N, 14^°^46′55″92 E), from a field developing at an altitude between 345 and 560 m above sea level. The area is characterized by temperate climate conditions. The soils of the area develop on lithotypes mainly formed by brown silt of pyroclastic origin with yellow sandy levels and by sand and heterogranular gravels (21).

Sampling was carried out over the period of ripeness, taking several samples for the same ripeness, to avert the results obtained for the same ripeness: green was sampled in the months of May to July, yellow in the months of September to mid-October, and orange and red from the second half of October to the beginning of December. A sample of 200 g was collected for each ripeness degree, to have sufficient sample to perform each type of analysis in triplicate. The samples of *A. unedo* fruits were sealed directly under vacuum in clean polyethylene bags and stored in refrigerated boxes at −4^°^C; they were taken to the laboratory within a maximum of 2 h of collection. Subsequently, all samples were left frozen at –24^°^C until they were analyzed and prepared manually.

### Preparation of *Arbutus unedo* L. Fruits’ Extracts

The samples of fruits with different ripeness were carefully washed with distilled water to remove impurities (dust and small insects). Each sample was chopped through the use of a mortar and recovered thanks to the use of a glass spatula.

A triplicate extraction was performed for each of the four ripening stages. The fruits were extracted with two different extraction techniques, maceration and sonic waves; in addition, two different solvents were used and their combinations: ethanol (EtOH), bidistilled water (H_2_O) (henceforth referred to as H1), and a solution of EtOH and H_2_O in two different ratios [7:3 and 3:7 (v:v)] (henceforth referred to as E7 and E3, respectively).

The extractions were conducted at 25.00 ± 1.00^°^C room temperature, at different times compared in the consideration of the same technique.

A list of the samples obtained and subjected to characterization can be found in Table 1. A unique code was associated with each sample.

**TABLE 1:** *A. unedo* fruit extract samples subjected to analysis.

Fruit	Extraction solvent	ID sample
Green	100% H_2_O	VH1
Green	30% EtOH in hydroalcoholic solution	VE3
Green	70% EtOH in hydroalcoholic solution	VE7
Yellow	100% H_2_O	GH1
Yellow	30% EtOH in hydroalcoholic solution	GE3
Yellow	70% EtOH in hydroalcoholic solution	GE7
Orange	100% H_2_O	AH1
Orange	30% EtOH in hydroalcoholic solution	AE3
Orange	70% EtOH in hydroalcoholic solution	AE7
Red	100% H_2_O	RH1
Red	30% EtOH in hydroalcoholic solution	RE3
Red	70% EtOH in hydroalcoholic solution	RE7

*V, green fruit; G, yellow fruit; A, orange fruit; R, red fruit; H1, extraction with 100% H2O; E7, extraction with 70% EtOH in hydroalcoholic solution; E3, extraction with 30% EtOH in hydroalcoholic solution.*

**FIGURE 1:**
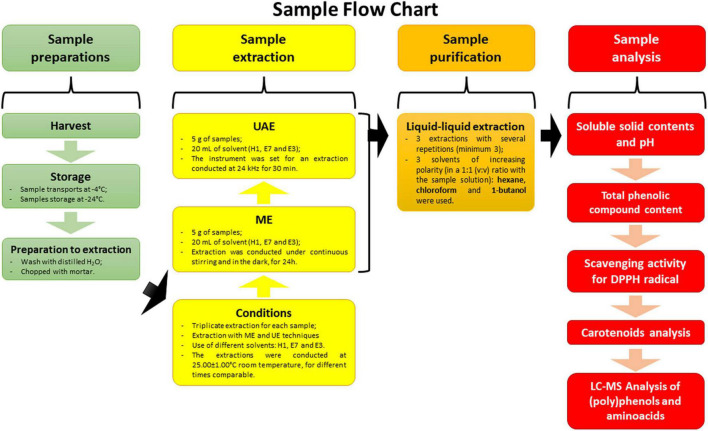
Graphical representation with sample flow chart: V, green fruit; G, yellow fruit; A, orange fruit; R, red fruit; H1, extraction with 100% H_2_O; E7, extraction with 70% EtOH in hydroalcoholic solution; E3, extraction with 30% EtOH in hydroalcoholic solution; ME, maceration extraction; UAE, ultrasound-assisted extraction; DPPH, 2,2-diphenyl-1-picrylhydrazyl radical.

A general outline of sample flow chart covering the preparation, extraction, purification, and analysis process is represented in Figure 1.

### Maceration Extraction

Extraction times through maceration extraction (ME) were chosen based on what was learned in the literature (22, 23) and the group’s experience with extractions. About 5 g of fruits was extracted by macerations with 20 mL of H_2_O and a solution of EtOH and H_2_O in two different ratios [7:3 and 3:7 (v:v)] under continuous stirring and in the dark, for 24 h. After maceration, the extracts were filtered through filter paper to remove any floating matter. After filtration, the clear solution extract obtained was stored in a dark container.

### Ultrasound-Assisted Extraction

The ultrasonic extractions were carried out by HEILSCHER ultrasound Technology (Teltow, Germany) UP200S and were equipped with an acoustic protection box. About 5 g of fruits was added to a flask with 20 mL and then placed in the chamber with the ultrasonic probe. The extractions were carried out under the same conditions with H_2_O and a solution of EtOH and H_2_O in two different ratios [7:3 and 3:7 (v/v)]. The instrument was set for an extraction conducted at 24 kHz and 25.00 ± 1.00^°^C for 30 min. The instrument uses a transducer attached to the probe that is immersed in the container including the solution with the sample: the action of the probe is direct, then the ultrasound generation in the middle decreases an extraction with minimal ultrasonic energy loss. In addition, the instrument is equipped with a thermostatic chamber in which the temperature of the sample is controlled by an infrared sensor, so that the increase in temperature of the sample generated by the action of the sonic waves is attenuated and controlled, thus avoiding damage to the thermolabile compounds. The instrument is generally operated at about 80% amplitude and with a continuous sonic wave solution interaction cycle. Each extraction cycle is 30 min long. After filtering the samples through filter paper, the obtained clear solution extracts were stored in a dark container.

### Extract Purification

To purify the target compounds obtained through the various extractions carried out, a liquid–liquid extraction protocol was performed using a separating funnel. The extractions involved the use of three extracting solvents of increasing polarity, used in sequence and with several repetitions of washes. Hexane, chloroform, and 1-butanol were used as solvents. The use of hexane and chloroform allowed to wash out the apolar compounds interfering with the detection of target analytes.

At least three washes were performed with each of the above solvents in a 1:1 (v:v) ratio with the sample solution. For each initial sample, then, three subfractions were obtained and analyzed, one for each extracting solvent. All solutions containing the extracted samples were subjected to the evaporation to calculate the yield. A rotary evaporator HEIDOLPH Heizbad Hei-Vap (Schwabach, Germany) was used to perform all sample desolvations, equipped with a 2L condensation chamber. All fractions where extracts were quantified were subjected to preliminary analysis: total phenolic content by Folin–Ciocâlteu reagent and scavenging activity with the DPPH method. Only the 1-butanol fractions resulted to be the richest in polyphenols and antioxidant compounds, so they were used to perform subsequent molecular characterization of *A. unedo* fruit at different ripening stages.

### Soluble Solid Contents and pH

Soluble solid content (°Bx) and pH were measured in all extracts by means of a refractometer and pH meter.

pH and temperature were determined by a CRISON (L’Hospitalet de Llobregat, Barcelona, Spain) GLP21 pH meter, a two-channel laboratory instrument.

A Brix and Gravity Refractometer with automatic temperature compensation (ATC) (with detection range of 0–32% Brix Grade and 1.000–1.130 for specific gravity, respectively) was used for specific gravity detection.

### Total Phenolic Compound Content

Total phenolic compound content was measured according to the Folin–Ciocâlteu reagent method (24). Briefly, 50 μL of sample extract was added to a cuvette. A quantity of 2,300 μL of bidistilled water and 50 μL of Folin–Ciocâlteu reagent were added, and after 6 min, 100 μL of sodium carbonate (Na_2_CO_3_) was added in the cuvettes. Each cuvette was shaken manually and allowed to stand for 90 min at room temperature. As the addition of sodium carbonate produces turbidity, which increases the absorbance signal, filtration of the solution was performed prior to absorbance measurement. Then, absorbance at 760 nm was measured, and the total phenolic compound content was expressed as gallic acid equivalents (GAE) as GAE concentration expressed in mol⋅L^–1^ using the calibration curve of gallic acid standard solutions (50–250 mg⋅L^–1^). All the measurements were taken in triplicate and calculated as a mean value ± *SD* (*n* = 3).

All absorbance measurements were taken by a MERCK (Milano, Italy) Spectroquant Pharo 300 UV/Vis spectrophotometer. A 1.0-cm-long optical path glass cell was employed in all measurements.

### Scavenging Activity for 2,2-Diphenyl-1-Picrylhydrazyl Radical

In the present experiment, the radical scavenging activity of extracts was estimated using the DPPH assay following the one used by Mosquera et al. (25), making some changes.

Each extract sample (50 μL) was solubilized in methanol (MeOH)–water solution (ratio 9:1 v:v). It was prepared 5 mM of DPPH in absolute ethanol. In the blank, 950 μL of MeOH and 50 μL of distilled water were added; in the control cuvette, 850 μL of methyl cyanide (CH_3_CN), 100 μL of DPPH 1 mM, and 50 μL of distilled water were added. In each cuvette, 850 μL of CH_3_CN, 100 μL DPPH 5 mM, and 50 μL of sample solution were added. The cuvettes were shaken manually and the absorption of the samples at 517 nm was spectrophotometrically determined at different time points (t_0_ to t_9_), over a period of 10 min with readings for every minute.

The radical scavenging activity was then expressed as the inhibition percentage of free radical by the sample and was calculated using the equation:


(1) $$\%\,D\,P\,P\,Hr\,a\,d\,i\,c\,a\,ls\,c\,a\,v\,e\,n\,g\,i\,n\,ga\,c\,t\,i\,v\,i\,t\,y=\,\left[\frac{\left(A_{c\,o\,n\,t\,r\,o\,l}-A_{s\,a\,m\,p\,l\,e}\right)}{A_{c\,o\,n\,t\,r\,o\,l}}\right]\times 100$$

where *A*_*sample*_ and *A*_*control*_ are the absorbance of the sample and the absorbance of the control solutions, respectively. Results were expressed as % free radical inhibition (I%). A stock solution with concentration 100 μg⋅mL^–1^ was prepared for each sample. For this concentration, the percentage ability of the substrate to reduce the absorbance of the radical DPPH available in the reaction medium was expressed. The I% is directly related to the antioxidant power of the sample.

### Liquid Chromatography Coupled to Mass Spectrometry Analysis

The 1-butanol extracts of *A. unedo* were performed by liquid chromatography coupled to mass spectrometry (LC-MS/MS) with multiple reaction monitoring mode (MRM), which serves as the basis for accurate simultaneous quantification of multiple analytes on sets of samples that generally come from extracts of natural matrices. Characterization of amino acids and (poly)phenols composition of the samples analyzed in triplicate by LC-MS/MS were named as follows: VH1, GH1, AH1, RH1, VE7, GE7, AE7, RE7, VE3, GE3, AE3, and RE3 (where V = green fruit, G = yellow fruit, A = orange fruit, R = red fruit, H1 = extraction with 100% H_2_O, E7 = extraction with 70% EtOH in hydroalcoholic solution, and E3 = extraction with 30% EtOH in hydroalcoholic solution).

LC Eksigent chromatographic system was coupled to a 4,000 QTRAP of AB SCIEX mass spectrometer equipped by ESI ion source. The chromatographic separation was performed on a C18 2.7 μm 90A 1 × 50 mm Halo column kept at 38^°^C to analyze amino acids and polyphenols.

Source-dependent parameters, ion spray voltage, source temperature, and nebulizer gas were set as 4.5 kV, 380^°^C, and 25 psi, respectively. The selection of the best precursor ion (Q1)- product ion (Q3) transitions in MRM ion mode and also the collision energy (CE) and declustering potential (DP) were previously reported by Pinto et al. (26), Lane (27), and Illiano et al. (28). The MRM analyses were performed in both positive and negative ion modes.

Skyline software 20.2.0.343 version (MacCoss Lab, Department of Genome Sciences, UW) was used for the interpretation of data.

1-Butanol extracts of *A. unedo* fruit were analyzed by mass spectrometry in multiple reaction monitoring ion mode. The methodology is greatly useful to the study of metabolic variations induced by genetic mutations or by dysfunctions of protein activity occurring in some pathologies (29, 30) or to monitor the levels of an analyte in biofluids included the absorption of some drugs. There are numerous studies demonstrating the versatility of the MRM/MS applications in food field not only for a molecular investigation of organoleptic and nutritional properties and of possible contaminants (31, 32) but also for the study of variations following particular treatments or different ripening stages (33–35).

#### Liquid Chromatography Coupled to Mass Spectrometry Chromatographic Conditions for Polyphenol Analysis

Polyphenols were separated by LC. Eluent A was H_2_O and 0.1% CH_3_COOH, whereas eluent B was a solution on 50% ACN, 50% isopropanol, and 0.1% CH_3_COOH. Gradient conditions were as follows: time 0 min, 20% eluent B; time 4 min 90% eluent B; time 4.5 min 20% eluent B; and time 5 min 20% eluent B. The injection volume was 5 μL, and the flow rate was fixed to 40 μL⋅min^–1^.

#### Liquid Chromatography Coupled to Mass Spectrometry Chromatographic Conditions for Amino Acid Analysis

Gradient conditions for amino acid analysis followed an increase of organic eluent from 0 to 90% in a 6-min run. Eluent A was an aqueous solution containing 10 mM ammonium formate and acidified with 0.1% formic acid, Eluent B 100% ACN containing 10 mM ammonium formate, and acidified with 0.1% formic acid.

### Data Analysis

Data were shown as mean and standard deviation (triplicate analyses). The one-way ANOVA was conducted to test group means and assess whether significant difference occurred between the extraction methods (at a *p-value* < 0.05). In addition, a two-dimensional PCA biplot was generated by PCA in R environment [R Core Team (36)] using the factoextra package [which provides ggplot2- based visualization (37)] after centered log-ratio (clr)-transformation of data according to compositional statistics (38, 39). PCA biplot was adopted to depict associations between samples and the analyzed compounds, which are expressed as dots and rays, respectively. The ray lengths are directly proportional to the variance of the corresponding variable included in the two components displayed, whereas the distance between two vectors depicts the link (correlation) between them (40).

## Results

The data obtained were processed and made explicit in tables in the following sections. In particular, the analyses performed through LC-MS with MRM allowed to provide high-quality information on target molecular profiles in a complex system: the profile of free amino acids and 56 molecules belonging to the large family of secondary metabolites of polyphenols was reported. A total of 19 out of 56 characterized compounds were found for the first time in *A. unedo*.

### Parameters of the Extracts

Extraction performance was evaluated on the extracts obtained: their weight was characterized, quantifying the dry residue, to evaluate both the extractive capacity of the solvent and the extraction techniques chosen. In addition, the extracted matrix samples were evaluated at the end of extraction, and their effective exhaustion in terms of total extract per amount of solvent used was verified. The values are shown in Table 2.

**TABLE 2:** pH, specific gravity, dry residue, and extract value of the different *A. unedo* fruit extracts.

Green fruit extracts	Maceration method				Ultrasonic method			
	pH	Brix [°Bx]	Residue (g⋅L^–1^)	Extract (g⋅g^–1^ F.W.)	pH	Brix [°Bx]	Residue (g⋅L^–1^)	Extract (g⋅g^–1^ F.W.)
H_2_O	6.67 ± 0.02	0.8 ± 0.1	21.20 ± 0.42	0.2092 ± 0.0011	6.44 ± 0.01	0.9 ± 0.1	31.84 ± 0.15 *	0.3405 ± 0.0021*
EtOH:H_2_O (7:3)	6.73 ± 0.01	1.3 ± 0.1	17.48 ± 0.02	0.1774 ± 0.0024	6.74 ± 0.01	1.0 ± 0.1	9.46 ± 0.12 *	0.09451 ± 0.00026*
EtOH:H_2_O (3:7)	6.71 ± 0.02	1.2 ± 0.1	6.42 ± 0.15	0.06420 ± 0.00080	6.84 ± 0.03	1.0 ± 0.1	21.30 ± 0.32 *	0.4057 ± 0.0010*

**Yellow fruit extracts**	**Maceration method**				**Ultrasonic method**			
	**pH**	**Brix [°Bx]**	**Residue (g⋅L^–1^)**	**Extract (g⋅g^–1^ F.W.)**	**pH**	**Brix [°Bx]**	**Residue (g⋅L^–1^)**	**Extract (g⋅g^–1^ F.W.)**

H_2_O	6.87 ± 0.01	1.0 ± 0.1	58.48 ± 0.25	0.5772 ± 0.0016	6.45 ± 0.02	1.1 ± 0.1	55.66 ± 0.21	0.5478 ± 0.00047
EtOH:H_2_O (7:3)	6.53 ± 0.01	1.4 ± 0.2	45.92 ± 0.16	0.4569 ± 0.0031	6.50 ± 0.01	1.3 ± 0.1	8.14 ± 0.03*	0.08036 ± 0.00013*
EtOH:H_2_O (3:7)	6.56 ± 0.02	1.4 ± 0.1	66.80 ± 0.11	0.6491 ± 0.0025	6.53 ± 0.02	1.2 ± 0.1	14.86 ± 0.32*	0.1466 ± 0.0010*

**Orange fruit extracts**	**Maceration method**				**Ultrasonic method**			
	**pH**	**Brix [°Bx]**	**Residue (g⋅L^–1^)**	**Extract (g⋅g^–1^ F.W.)**	**pH**	**Brix [°Bx]**	**Residue (g⋅L^–1^)**	**Extract (g⋅g^–1^ F.W.)**

H_2_O	6.62 ± 0.03	0.9 ± 0.1	38.06 ± 0.40	0.3806 ± 0.0012	6.60 ± 0.02	1.1 ± 0.1	11.66 ± 0.04*	0.1157 ± 0.0031*
EtOH:H_2_O (7:3)	6.68 ± 0.02	1.3 ± 0.1	45.98 ± 0.27	0.4561 ± 0.0018	6.75 ± 0.01	1.3 ± 0.2	33.06 ± 0.18*	0.3306 ± 0.0023*
EtOH:H_2_O (3:7)	6.65 ± 0.01	1.2 ± 0.1	44.54 ± 0.14	0.4431 ± 0.0020	6.81 ± 0.01	1.1 ± 0.1	32.02 ± 0.34*	0.3225 ± 0.0026*

**Red fruit extracts**	**Maceration method**				**Ultrasonic method**			
	**pH**	**Brix [°Bx]**	**Residue (g⋅L^–1^)**	**Extract (g⋅g^–1^ F.W.)**	**pH**	**Brix [°Bx]**	**Residue (g⋅L^–1^)**	**Extract (g⋅g^–1^ F.W.)**

H_2_O	6.53 ± 0.02	1.2 ± 0.1	47.00 ± 0.22	0.4667 ± 0.0054	6.75 ± 0.02	1.0 ± 0.1	13.72 ± 0.03*	0.1366 ± 0.00023*
EtOH:H_2_O (7:3)	6.51 ± 0.04	1.5 ± 0.2	58.12 ± 0.12	0.5806 ± 0.0061	6.63 ± 0.02	1.4 ± 0.2	75.42 ± 0.26*	0.7504 ± 0.0025*
EtOH:H_2_O (3:7)	6.68 ± 0.01	1.3 ± 0.2	72.50 ± 0.28	0.7100 ± 0.0049	6.76 ± 0.02	1.1 ± 0.1	79.64 ± 0.38*	0.7838 ± 0.0014*

*F.W., fresh weight.*

*indicate significant differences for parameters between the two extraction methods at p < 0.05.

*Values are presented as mean (triplicate) ± SD.*

Overall, as it is possible to observe from the data reported in Table 2, important differences exist between the two extraction techniques used both with regard to the dry residue and to the total extract obtained, whereas no significant differences are observed with regard to pH and Bx°.

### Total Phenolic Compound Content

The total phenolic content was analyzed in all samples, but only 1-butanol samples showed a result. The total phenolic compound content (TPC) of the different *A. unedo* fruit extracts is shown in Table 3.

**TABLE 3:** Total phenolic compound content (TPC) of the different *A. unedo* fruit extracts.

	Total phenolic compound content [*C* GAE⋅(mol⋅L^–1^)]					
	Maceration method			Ultrasonic method		
	H_2_O	EtOH:H_2_O (7:3)	EtOH:H_2_O (3:7)	H_2_O	EtOH:H_2_O (7:3)	EtOH:H_2_O (3:7)
Green	0.2735 ± 0.0029	0.3553 ± 0.0090	0.2489 ± 0.0090	0.3033 ± 0.0037*	0.3530 ± 0.036	0.2760 ± 0.013*
Yellow	0.3233 ± 0.0097	0.3558 ± 0.033	0.3238 ± 0.0057	0.3458 ± 0.0014*	0.3598 ± 0.0092	0.3253 ± 0.0009
Orange	0.3375 ± 0.0095	0.3620 ± 0.0058	0.3415 ± 0.0078	0.3735 ± 0.057*	0.3628 ± 0.0070	0.3455 ± 0.0092
Red	0.3455 ± 0.026	0.3670 ± 0.0019	0.3490 ± 0.0022	0.3558 ± 0.043	0.3640 ± 0.0075	0.3483 ± 0.0018

*GAE expresses the equivalents of gallic acid.*

*indicate significant differences for parameters between the two extraction methods at p < 0.05.

Regarding TPC, it is possible to show a significant difference between the two methods in all ripening stages when H_2_O is the extraction solvent. Only for the extract of the green fruit, obtained both with UAE and with EtOH:H_2_O [in a ratio of 3:7 (v:v)], there was a difference in consideration of TPC (Table 3). Even if the results show a very good yield in TPC, it must be taken into account that the content is overestimated because the extracts in 1-butanol may contain different molecules (as in the case of proteins and amino acids, the latter analyzed in this work) that can respond positively to the assay and generate a consequent overestimation.

### Radical Scavenging Activity

Values correlated at the DPPH radical scavenging activity (I%) of the different *A. unedo* fruit extracts are given in Table 4. The I% was calculated in all samples, but, as for TPC, only the 1-butanol samples showed a result, which was reported. Overall, I% values highlight the significant differences between the two extraction methods for all solvents and all ripening stages.

**TABLE 4:** DPPH radical scavenging activity (I%) of the different *A. unedo* fruit extracts.

	% Free radical inhibition (I%)					
	Maceration method			Ultrasonic method		
	H_2_O	EtOH:H_2_O (7:3)	EtOH:H_2_O (3:7)	H_2_O	EtOH:H_2_O (7:3)	EtOH:H_2_O (3:7)
Green	40.51 ± 0.84	57.14 ± 17.14	48.98 ± 13.73	64.84 ± 2.61*	66.87 ± 0.55*	63.54 ± 1.19*
Yellow	61.32 ± 3.54	64.64 ± 0.29	58.09 ± 2.98	80.27 ± 0.88*	85.24 ± 0.65*	79.23 ± 0.89*
Orange	56.96 ± 0.69	57.08 ± 2.53	51.28 ± 0.95	66.08 ± 0.57*	74.32 ± 0.34*	65.92 ± 0.53*
Red	57.60 ± 0.53	61.81 ± 0.59	56.37 ± 1.25	78.40 ± 0.91*	83.57 ± 1.89*	77.62 ± 0.68*

*indicate significant differences for parameters between the two extraction methods at p < 0.05.

*Values are presented as mean (triplicate) ± SD.*

### Bioactive Compounds Profile Under Different Ripening by Liquid Chromatography Coupled to Mass Spectrometry Analysis

Here, we presented the application of mass spectrometry in MRM mode for the qualitative-quantitative monitoring of free amino acids (Table 5) and 56 molecules belonging to the large family of secondary metabolites of polyphenols (Table 6). Among all selected polyphenols, 19 were identified for the first time in *A. unedo* (compounds are marked with a * in Table 6).

**TABLE 5:** Amino acids identified in *A. unedo* fruit extracts by LC-MS/MS analysis (expressed as percentage, %).

Amino acid	VH1	GH1	AH1	RH1	VE7	GE7	AE7	RE7	VE3	GE3	AE3	RE3
Alanine	4.61	8.17	9.52	6.15	1.30	n.q.	n.q.	n.q.	4.01	3.97	8.76	7.76
Valine*	2.54	4.59	3.65	2.60	0.58	3.51	8.49	5.51	15.58	14.43	4.12	4.07
Leucine*	0.68	0.58	0.17	0.14	0.23	5.62	3.32	4.63	1.07	1.02	0.23	0.44
Isoleucine*	11.95	11.64	n.q.	n.q.	6.64	3.77	4.27	8.83	22.36	21.15	12.52	10.89
Tryptofano*	40.64	2.80	3.59	4.68	72.29	2.71	5.20	7.96	2.50	2.33	7.28	3.08
Tyrosine	4.63	14.23	12.08	18.10	4.90	11.89	9.21	15.40	8.42	8.59	10.97	16.06
Phenylalanine*	0.25	0.18	0.17	0.25	0.13	3.51	7.41	10.20	0.17	0.22	0.64	0.45
Aspartic acid	10.31	18.76	23.59	18.18	3.34	8.38	4.88	2.88	21.25	22.02	22.24	19.90
Asparagine	17.12	26.49	34.71	39.96	8.74	7.76	4.48	2.97	13.26	14.36	21.22	25.50
Glutamine	0.57	n.q.	n.q.	n.q.	0.07	1.78	2.95	1.02	n.q.	n.q.	n.q.	n.q.
Glutamic acid	3.27	5.40	5.33	4.20	0.78	10.27	12.18	10.47	4.61	5.03	4.20	4.68
Arginine	0.43	n.q.	0.11	0.13	0.04	1.42	0.23	0.45	0.05	0.07	0.15	0.17
Lysine*	1.04	n.q.	n.q.	n.q.	0.11	1.93	2.92	1.96	n.q.	n.q.	0.26	0.00
Histidine*	0.32	0.16	0.48	0.31	0.06	2.41	1.10	0.86	0.10	0.11	0.39	0.37
Serine	1.66	5.14	5.19	3.18	0.62	2.82	1.26	1.73	5.28	5.37	5.03	4.39
Threnonine*	n.q.	n.q.	n.q.	n.q.	n.q.	30.67	30.52	23.78	n.q.	n.q.	n.q.	n.q.
Cysteine*	n.q.	n.q.	n.q.	n.q.	n.q.	n.q.	n.q.	n.q.	n.q.	n.q.	n.q.	n.q.
Methionine*	0.00	1.84	1.41	2.13	0.16	1.56	1.58	1.34	1.34	1.34	1.99	2.23

n.q., not quantifiable; *essential amino acids.

**TABLE 6:** Metabolites identified in *A. unedo* fruit extracts by LC-MS/MS analysis (expressed in ng⋅g**^–^**^1^ of FW).

Analyte	VH1	GH1	AH1	RH1	VE7	GE7	AE7	RE7	VE3	GE3	AE3	RE3
Delphinidin-3-O-glucoside	21.16	52.74	99.66	100.34	1929.3	680.62	649.3	1101.9	836.9	561.14	309.44	1063.5
Delphinidin-3-O-arabinoside	7	12.72	11.6	17.2	774.8	440.96	341.22	720.36	431.76	276.14	227.72	719.02
Delphinidin rutinoside*	79.32	20.34	14.18	25.48	152.92	42.64	43.14	49.46	91.56	64.08	33.48	58.68
Delphinidin diglucoside*	43.42	28.36	23.6	134.84	71.42	53.68	99.66	153.18	102.74	69.8	55.24	157.02
Cyanidin-3-O-glucoside	8	23.52	30.02	291.16	173.78	308.12	1441.72	4823.74	260.58	164.12	251.82	5670.5
Cyanidin-3-O-arabinoside	6.44	9.06	8.18	43.04	131.14	38.18	197.84	1054.5	98.14	63.12	115.38	858.38
Cyanidin-3-5-di-O-glucoside	10.12	7.84	15.78	410.66	7.44	12.02	179.76	798.5	35.82	22.56	28.92	731.44
Malvidin-3-O-glucoside	17.38	179.5	109.76	69.76	234.28	51.64	70.74	52.72	468.44	309.4	140.44	195.78
Malvidin-3-O-arabinoside*	7.68	9.76	7.3	9.72	49.52	9.6	8.46	8.2	95.74	65.52	61.54	41.74
Malvidin 3-O-p-coumaroylglucoside*	11.8	20.38	17.56	20.7	n.q.	9.3	27.18	17.56	0.08	n.q.	n.q.	n.q.
Petunidin-3-O-glucoside*	20.14	52.02	49.78	44.02	14.46	87.92	80.58	65.72	47.18	31.14	43.48	33.82
Petunidin-3-O-arabinoside*	45.58	6.98	16.42	37.96	n.q.	36.86	7.66	74.26	95.86	60.44	52.08	7.76
Peonidin-3-O-glucoside	341.44	25.52	122.54	299.18	18.98	285.5	63.12	182.9	308.54	218.9	155.7	155.56
Pelargonidin-3-O-glucoside	44.5	188.94	123.62	204.34	68.1	145.68	165.1	177.5	271.74	170.14	198.66	200.94
Epicatechin	189.54	168.7	282.44	5865.98	2928.36	1633.14	1033.54	41119.76	12937.68	9021.34	1051.5	14156
Procyanidin B1	211.12	21.1	39.18	127.96	298.4	32.64	38.92	386.68	128.4	83	81.82	206.26
Procyanidin C*	9.72	38.14	27.84	26.2	14	22.62	18.84	28.94	21.04	14.38	18.6	21.52
Procyanidin tetramer	20.56	13.94	10.52	31.26	9.84	50.62	40.14	77	58.4	40.1	50.48	69.28
Procyanidin pentamer	n.q.	n.q.	n.q.	n.q.	n.q.	n.q.	n.q.	n.q.	0.1	n.q.	n.q.	n.q.
Catechin-3-gallate*	47.5	173.54	111.74	127.82	131.78	179.52	131.02	124.06	215.2	152.04	166.32	91.86
Gallocatechin	53.98	30.72	46.48	778.64	199.2	228.08	154.96	2197.7	763.78	527.7	107.24	1055.5
EC-3-gallate	44.44	177.12	101.64	115.12	142.92	186.5	131.72	120.32	264.1	171.44	133.84	73
EGC 3-gallate	61.98	20.04	14.32	11.46	114.58	90.06	24.54	64.06	103.62	65.32	46.58	46.12
GC 3-gallate	64.78	17.54	12.28	11.46	120.66	34.86	26.98	65.58	125.24	82.02	73.04	62.16
EGC-epicatechin dimer	24.26	15.48	14.84	118.08	25.32	25.04	15.92	303.14	136.5	94.32	35.82	152.18
EGC digallate*	n.q.	n.q.	n.q.	n.q.	n.q.	n.q.	22.16	74.68	75.22	47.28	34.56	50.52
EGC gallate glucoside*	61.92	69.56	42.32	59.86	n.q.	17.44	15.98	14.24	9.46	6.72	n.q.	n.q.
EGC-EGC gallate*	n.q.	n.q.	n.q.	n.q.	n.q.	n.q.	n.q.	n.q.	10.28	6.72	n.q.	n.q.
Flavone+Na	n.q.	n.q.	n.q.	n.q.	138.8	211.38	263.8	76.4	135.46	92.14	130.28	99.96
Apigenin	1014.62	415.2	406.38	688.5	842.96	944.3	545.52	752.94	896.72	574.82	846.08	644.34
Daidzein*	243.32	87.8	58.72	90.66	174.28	73.76	89.74	41.68	41.22	28.84	42.24	44.08
Eriodictyol	470.64	406.42	500.56	745.98	347.2	1228.12	1479.64	1832.94	791.34	507.38	503.54	549.16
Quercetin	2256.12	748.68	954.82	1093.84	4377.12	2312.64	2025.3	4418.02	2599.74	1730.4	1092.28	2647.2
Quercetin-3-glucoside	583.24	245.76	237.98	781.52	2053.4	726.58	526.86	999.8	962.06	643.18	270.64	1102
Sinensetin*	n.q.	n.q.	n.q.	n.q.	n.q.	101.62	71.34	62.98	0.1	n.q.	n.q.	n.q.
Naringin*	181.06	165.28	103.98	245.52	243.56	231.42	270.92	252.82	330.5	226.8	171.98	244.84
Naringenin*	571.44	375.4	241.92	696.42	n.q.	857.06	773.84	470.84	0.1	n.q.	n.q.	n.q.
Myricetin	21.76	14.96	13.6	19.76	15.76	15.86	14.42	11.14	16.86	10.54	10.62	11.84
Myricitrin*	701.5	2012.96	2545.74	1887.44	381.04	645.34	1244.96	879.08	1754.72	1182.38	3067.92	836.8
Kaempferol	307.42	291.12	859.28	226.6	312.94	427.26	217.24	170.18	202.62	132.98	172.1	141.64
Phloridzin	385.94	183.56	161.48	736.9	619.88	1224.22	698.02	1272.3	1340.08	902.68	360.78	1057.7
Phloretin*	325.98	181.42	168.78	171.62	188.98	195.66	168.34	153.42	264.72	178.92	169.06	158.32
Rutin	71.16	20.72	12.6	29	152.08	42.04	43.34	55.74	89.22	60.66	34.56	45.6
6-Malonyldaidzin*	132.36	58.06	44.02	58.56	91.26	158.68	31.1	106.56	130.32	86.54	79.78	84.22
Gallic acid	34439.12	11602.52	16947.96	19119.32	23127.86	9538.06	5372.48	3749.6	17082.86	11205.44	10050.62	4966.6
Syringic acid	865.96	1233.92	1017.18	1283.42	2166.44	2155.54	1563.04	813.06	3741.38	2600.3	2434.12	1305.6
Chlorogenic acid	345	644.24	409.98	639.1	310.32	768.92	992.94	389.12	837.4	597	543.22	512.6
Caffeic acid	394.38	211.94	193.62	187.96	199.46	181.3	219.26	167.94	286.86	185.76	187.84	171.46
Quinic acid	1136.84	1593.3	1015.3	1602.3	1063.82	2838.98	2314.14	1060.64	1889.62	1331.22	1241.52	1116.7
Ferulic acid	4750.72	2102.8	1520.4	2356	2053.02	2353.94	2345.74	764.42	3329.78	2166.84	2005.44	1162.3
Coumaric acid	n.q.	n.q.	n.q.	n.q.	n.q.	n.q.	n.q.	n.q.	227.2	143.02	n.q.	n.q.
Vanillic acid	759.6	225.1	191.54	263.12	567	373.76	262.94	205.64	507.7	324.64	319.1	217.3
Coumaroylquinic acid	299.2	477.32	278.26	342.94	165.86	214.2	409.16	215.16	227.58	143.02	n.q.	205.72
Feruloylquinic acid	234.7	247.62	171.06	168.8	201.82	189.54	181.96	151	287.1	197.56	190.4	167.06
Valoneic acid dilactone*	43.84	29.18	27.92	26.74	18.04	22.56	22.28	14.92	27.7	17.46	20.9	16.48
Caffeine	8.54	n.q.	n.q.	0.78	n.q.	3.84	3.52	n.q.	11	7.5	2.08	7.94

n.q., not quantifiable. *compounds identified for the first time in A. unedo.

The MRM ion mode allows the identification of target analytes based on the specific precursor ion to product ion transitions. The proposed methodology provides the two or three best transitions for each molecule ensuring greater specificity and selectivity. The transitions were selected, for each analyte, thanks to an in-depth study of the literature and the analysis of fragmentation spectra obtained in our previous studies (26, 28, 41).

**FIGURE 2:**
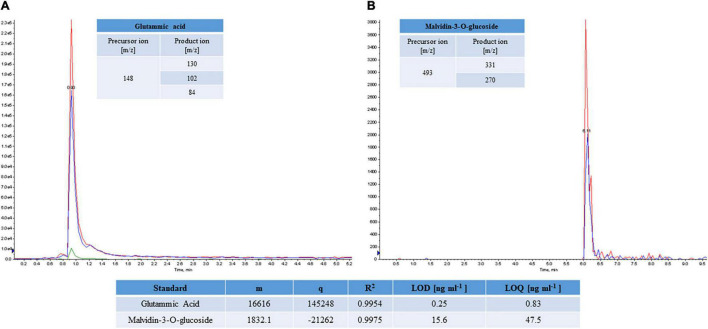
MRM chromatograms of glutamic acid **(A)** and malvidin-3-O-glucoside **(B)** standard molecules. The best transitions 148 m/z→130 m/z, 148 m/z →102 m/z and 148 m/z →84 m/z and 493 m/z →331 m/z and 493 m/z →270 m/z were monitored for glutamic acid and malvidin-3-O-glucoside, respectively. Analytical parameters calculated for the standards (LOD, LOQ), angular coefficient (m), y-intercept (q), and *R*^2^ are reported.

The limited availability of pure standards did not enable to build up calibration curve for each molecule; thus, the quantitative analysis was carried out by exploiting the structural and fragmentation homology of the similar molecules. Only eight polyphenol standards and the amino acid mixture were used to realize the calibration curves in a linear range of concentration (0.1–500 ng⋅mL^–1)^. As an example, the Figure 2 reported the MRM chromatogram of glutamic acid and malvidin-3-O-glucoside. Moreover, the calculated analytical parameters, e.g., limit of detection (LOD), limit of quantification (LOQ), the angular coefficient, y-intercept, and *R*^2^ were included in the figure.

The butanol extracts of *A. unedo* were analyzed by LC-MS/MS in triplicate. By importing the.wiff files on the Skyline software, the identification of each target molecules was assigned by monitoring the perfect co-elution of the selected transitions. The peak areas of transitions were recorded for all replicates, and the calculated coefficient of variation (CV%), ranging from 5 to 20%, was used to assess the good reproducibility and repeatability of the method. For the quantitative analysis, the peak areas were interpolated on calibration curves accordingly to structural homology, to obtain the compound concentration. The polyphenol content was reported in Table 6 as ng⋅g^–1^ of FW (Table 6), while those of amino acids was expressed as % on the total amount of all amino acids (Table 5).

### Bioactive Compounds Profile Under Different Ripening Stages

LC-MRM/MS data collected were then processed using multivariate data analysis. With the aim of performing a comparative study of the extracts of *A. unedo*, the PCA approach was applied in an untargeted way to acquired raw data.

**FIGURE 3:**
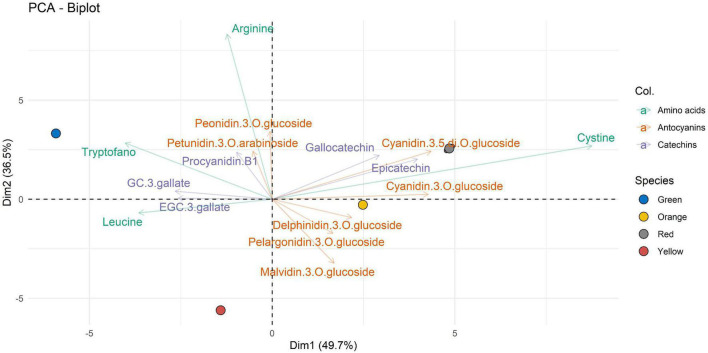
Two-dimensional principal component analysis (PCA) biplot showing associations between experimental samples (*A. unedo* fruit) and compounds abundance (clr-transformed data). Original data are summarized in Table 6 and in Supplementary Table 1. Compounds extracted in H1 are represented. Samples are shown according to the ripening stages (green, yellow, orange, and red) and compounds are shown according to polyphenol categories.

**FIGURE 4:**
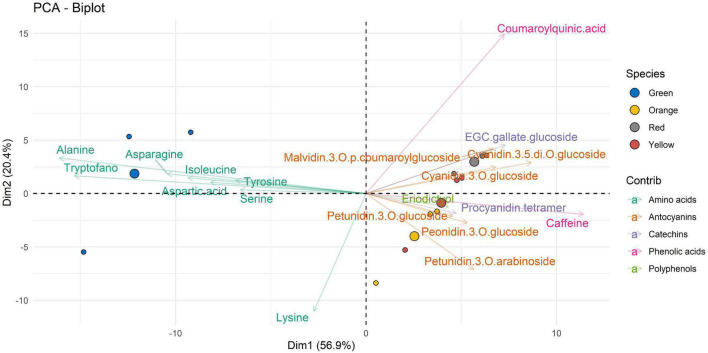
Two-dimensional principal component analysis (PCA) biplot showing associations between experimental samples (*A. unedo* fruit) and compounds abundance (clr-transformed data). Original data are summarized in Table 6 and in Supplementary Table 1. Compounds extracted in E3 are represented. Samples are shown according to the ripening stages (green, yellow, orange, and red) and compounds are shown according to polyphenol categories.

The PCA biplots (Figures 2–4) were produced with the first two components (PC1 and PC2), to interpret the maximum differences and unveil the relationships between ripening stage and extracted bioactive compounds, by the three different types of solvent used for the extraction of the compounds of interest in this work. For values expressed as not quantifiable, the minimum value below which the analyte cannot be quantified, i.e., 0.3 ng⋅mL^–1^, was assigned in the graphical data processing.

In Figure 3, where the extraction in H1 is represented, the first 2 principal components (displayed as Dim1 and Dim2, respectively) account for 86.2% of total variability. The first principal component (Dim1), representing the maximum variance direction in the data, accounted for 49.7% of the overall variability. High scores on Dim1 allow to distinguish two main clusters of compounds.

It is possible to observe a first group of some well-characterized amino acids in the green samples. In contrast to the amino acids represented for the green ripening state, there is cystine, which from the characterization data appears well represented in more advanced ripening states such as orange and red. The catechins characterized are represented through three of the four ripening states (green, orange, and red): specifically, gallocatechin and epicatechin are well represented in the orange and red ripening states, whereas procyanidin B, GC 3-gallate, and EGC 3-gallate are represented in green. Anthocyanins, which are usually well represented in the more advanced stages of ripeness, can be seen as being present in the orange and red ripening stages. Peonidin-3-O-glucoside and petunidin-3-O-arabinoside detach from this representation, and both are represented in the green ripening state and related to arginine (as shown by the second component).

Figure 4 shows the data of the molecular compounds with respect to the samples extracted with E3. Looking at Dim1 (which account for 56.9% of the overall variability), it is possible to observe how the amino acids such as alanine, tryptophane, asparagine, aspartic acid, isoleucine, and serine dominate the metabolite profile of the green ripeness. The other compounds, such as anthocyanins, catechins, phenolic acids, and polyphenols, in general, are well represented by the other states of maturation. It is important to note that these compounds are antithetical to those of the amino acids. Dim2, accounting for 20.4% of variability, is featured by an opposite correlation between lysine and coumaroylquinic acid. This opposite behavior would deserve attention in the future studies.

**FIGURE 5:**
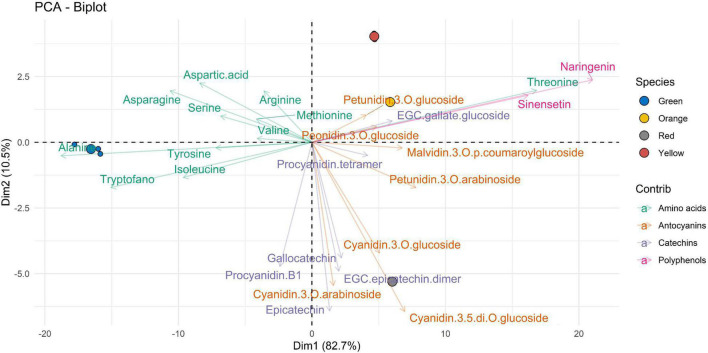
Two-dimensional principal component analysis (PCA) biplot showing associations between experimental samples (*A. unedo* fruit) and compounds abundance (clr-transformed data). Original data are summarized in Table 6 and in Supplementary Table 1. Compounds extracted in E7 are represented. Samples are shown according to the ripening stages (green, yellow, orange, and red) and compounds are shown according to polyphenol categories.

Finally, Figure 5 shows the data of the molecular compounds with respect to the samples extracted with E7. Also, in this figure, in the Dim1 (accounting for 82.7% of variability), the amino acids are well clustered according to the green ripening state, except for threonine which has an opposite behavior. Polyphenols (such as naringenin and sinensetin) and threonine are well correlated according to yellow and orange ripeness. The Dim2 (accounting for 10.5%) suggests that the profile of bioactive compounds of the most advanced ripening stage (red) is characterized by a predominance of catechins (such as gallocatechin, epicatechin, procyanidin B1, and EGC–epicatechin dimer) and anthocyanins (such as cyanidin-3-O-glucoside, cyanidin-3-O-arabinoside, and cyanidin-3-5-di-O-glucoside).

## Discussion

Given the important variations during the ripening of the strawberry tree fruit, and the fragmentary information in the literature, a phytochemical characterization was carried out on the fruits in four ripening stages (Green/Yellow/Orange/Red). This may be fundamental for understanding the evolution of the bioactive compounds produced and present within the fruit, understanding how they evolve according to the state of ripeness and may open the way to new studies from a metabolomic and proteomic point of view. Furthermore, to evaluate which solvent/extraction technique combination performs best on this matrix, two types of extractions and three solvents were tested.

### Evaluation of Extract Parameters

An important result (expressed in Brix degrees) was obtained in the evaluation of the soluble solid. These data (°Bx), obtained through a first quantification of all extracts and considering the specific weight of each extract (extract value express in grams of extract per grams of fresh fruit weight), are very low when compared to the large amount of all possible components present in the extract. This result would suggest that in the analysis of the content of total phenolic compounds, developed subsequently, the value expressed in total extract weight is almost entirely due to the presence of (poly)phenols and similar compounds.

### Total Phenolic Compound Content Evaluation

The data obtained by comparing the different degrees of ripeness do not show significant differences, expressed in TPC, compared to the different ripeness stages. For the green ripening stage only, lower results were obtained (in the order of 10^–2^ M) than the other ripening states with the same extractive solvent and extraction technique used. The TPC of extracts, estimated by the Folin–Ciocâlteu reagent method, varies between the two extraction techniques, maceration, and ultrasound.

The total yield of the phenolic compound in the EtOH:H_2_O mixture at a ratio of 7:3 (v:v) (E7) was higher (although in some cases in the order of 10^–2^–10^–3^ M) than that of the other solvents for both extraction methods. This result has shown that the mixture EtOH:H_2_O, both mixture at a ratio of 7:3 (v:v) and mixture at a ratio of 3:7 (v:v) (E3), with a higher percentage of EtOH than H_2_O, is the most suitable solvent for the phenolic compound extraction, and TPC is improved by increasing the polarity of the solvent used to purify the initial extract, regardless of the extraction method. As regards the extraction method, the TPC in *A. unedo* fruit extracted with ME and UAE was substantially the same. It should be noted that extraction with the UAE is certainly a type of extraction that is conducted in a shorter time but, when compared to other methods such as ME, has a definitely higher energy cost. These data therefore, show that the phenolic compound content of *A. unedo* extracts is not only influenced by the polarity of the solvent but also by the extraction process and method. Following on from this discussion, it is interesting to see that the performance of TPC extractions is almost constant across the different ripening stages. This could probably be due to a solvent saturation in the extraction of these compounds, further studies should be undertaken following only the solvent/matrix ratio during the extraction process.

### Evaluation of Radical Scavenging Activity

As it is possible to observe from the data, comparing the ripening stages, the yellow and the red fruit stages showed higher DPPH activity than the green one. Ripening is a determining process as regards the phytochemical content of the fruit, and it is well known how compounds with antioxidant activity accumulate in the ripening fruit (42), in fact, not only from the point of view of palatability, but also nutraceutical, the consumption of strawberry tree fruit in the advanced stages of ripeness is preferable.

The data in Table 4 show a slight preference for the extraction of anti-radical molecules with the UAE over the ME. Most likely, this result is due to the longer extraction time for ME (24 h), which means a higher probability for molecules with anti-radical activity to react with air or be subjected to degradation by light and heat, even when working under stable and safe conditions.

The total antioxidant activity of extracts from natural matrix depends on both the total content of phenolic compounds and the presence of chemical classes other than phenolic compounds that can react with the DPPH^⋅^ radical. On the other hand, extracts obtained with different solvents and methods include different minor components that may, in relation to the action of a synergistic behavior with other components, have a greater effect on the antioxidant capacity, thus differing from extract to extract.

The acquired data show a good anti-radical activity of the extracts produced with both techniques, with preference for extraction with UAE over ME.

### Evaluation of Liquid Chromatography Coupled to Mass Spectrometry Analysis

The MRM-based methodology is greatly useful to study the metabolic variations induced by genetic mutations or by dysfunctions of protein activity occurring in some pathologies (29, 30) or to monitor the levels of an analyte in biofluids. There are numerous studies demonstrating the versatility of the MRM/MS applications in food field not only for a molecular investigation of organoleptic and nutritional properties and of possible contaminants (31, 32) but also for the study of metabolomic variations following particular treatments or different ripening stages (33–35).

Tables 5, 6 collected the data obtained by MRM/MS analysis on fruit samples in the different ripening stages treated with 100% H_2_O, 7:3 ratio EtOH: H_2_O, and 3:7 ratio EtOH:H_2_O (E7, E3, and H1).

Analysis of the polyphenol fraction was carried out by grouping the target molecules into four main subgroups according to their structures such as anthocyanins, catechins, phenolic acids, and other flavonoids which mainly belong to flavan-3-ols.

**FIGURE 6:**
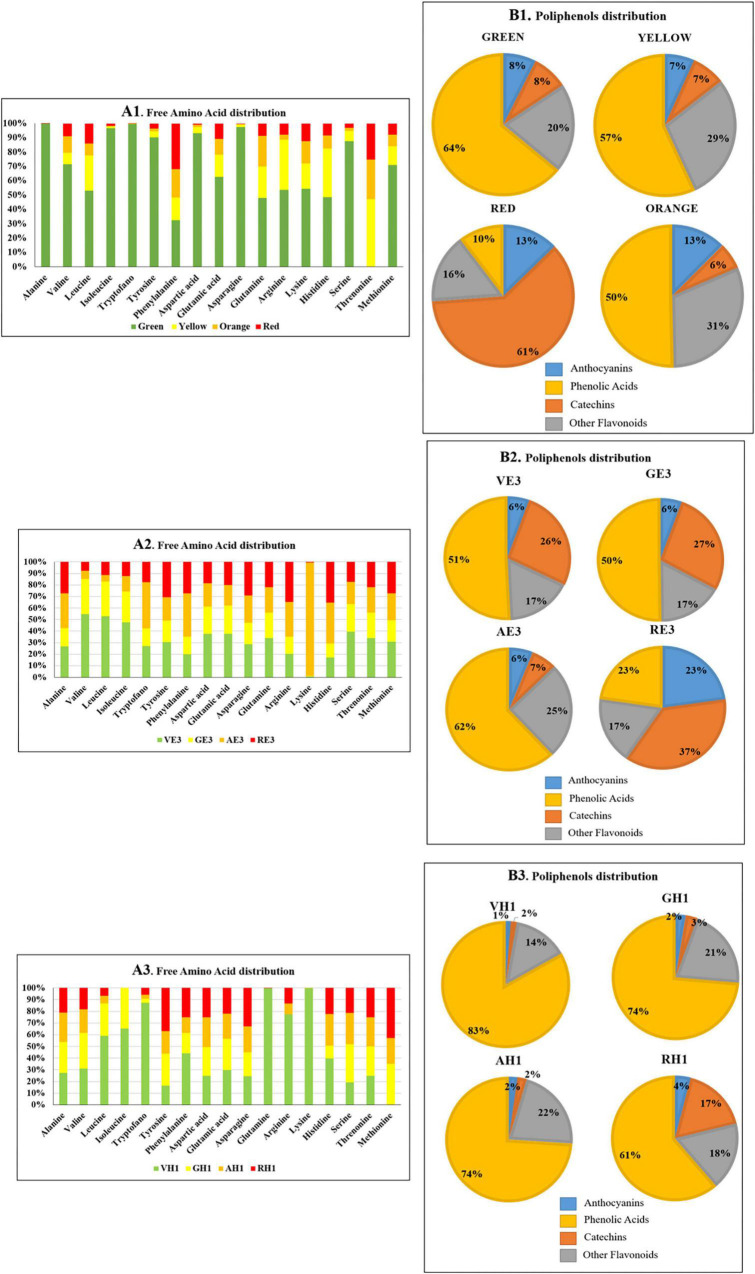
Summary of MRM/MS E7, E3, and H1 analysis of butanol extracts of *A. unedo* fruit samples in different ripening stages (green, yellow, orange, and red) **(A1)** free amino acid distribution. **(B1)** Pie charts pointing the percentage composition of *A. unedo* polyphenolic fraction extracted with the mixture E7. Four different compound classes were grouped: anthocyanins, phenolic acids, catechins, and other flavonoids. **(A2)** free amino acid distribution. **(B2)** Pie charts pointing the percentage composition of *A. unedo* polyphenolic fraction extracted with the mixture E3. A total of four different compound classes were grouped: anthocyanins, phenolic acids, catechins, and other flavonoids. **(A3)** Free amino acid distribution. **(B3)** Pie charts pointing the percentage composition of *A. unedo* polyphenolic fraction extracted with the mixture H1. A total of four different compound classes were grouped: anthocyanins, phenolic acids, catechins, and other flavonoids.

Panels A1, A2, and A3 in Figure 6 reported the distribution of free amino acids expressed as percentage on the total amount of amino acids in the four ripening stages and considering the three extraction solvents E7, E3, and H1, respectively. In general, the content of free amino acids decreases from green to red ripening state. In *A. unedo* green fruit, there is the higher content of amino acids with the only exception of phenylalanine and threonine. This aspect is particularly highlighted in Figure 6 Panel A1. In agreement with studies carried out on other plants (43–45), the accumulation of amino acids in the early stages of ripening is due to the future biosynthesis of bioactive chemical compounds, small peptides, and proteins that will be produced within the fruit as it matures. The distribution of the free amino acid content is different in the three extracting solvents.

In Figure 6, Panels A1, A2, and A3 highlight that more amino acids in the green fruit stage were extracted in E3 than in E7 and H1, in particular alanine, isoleucine, tryptophan, tyrosine, asparagine, and serine. In the E7 orange ripening stage, there is the highest abundance of lysine. Finally, in H1, the extraction of lysine, arginine, glutamine, and tryptophan in the *A. unedo* green ripening stage is favored.

Although the anthocyanin content does not vary significantly with the maturation, the level of some individual molecules changes a lot, and examples are cyaniding-3-glucoside whose concentration ranges between 179 and 4,824 ng⋅g^–1^ from green to red. This molecule is normally associated with the red color of many fruits such as plums, grapes, acai berries, and so on (46). And its trend of expression is in agreement with the change in color of *A. unedo* during ripening.

A progressive decrease in phenolic acids (64% in green and 10% in red) and an increase in catechin content (from 8% in green to 61% in red) were observed with the fruit ripening for E7 extraction (Figure 6 Panel B1). The same trend for phenolic acids was observed for E3 (51% in green and 23% in red) and H1 (83% in green and 61% in red) extracts during the ripening as reported in Figure 5 Panel B2 and Panel B3, respectively. However, the analyses revealed a similar extraction level using hydroalcoholic solvents for anthocyanins (Figure 6 Panel B1, Panel B2, and Panel B3) except for RE3. On the other hand, differences in the phenolic acids and catechins content could be detected. As an example, AE7 shows a higher extraction level of catechins than AE3, whereas the reverse is true for phenolic acids. By comparing the relative percentage of the other flavonoids, no differences were observed between E7 and E3. Surprisingly, H1 and E7 showed a similar relative percentage of the various components, except for AH1 displaying a relative percentage of the components comparable to AE3.

Many compounds found and identified had already been reported in *A. unedo*. Some were reported for the first time. The main metabolites detected are anthocyanins, catechins, phenolic acids, and polyphenols, in general. The anthocyanins found corresponded mainly to delphinidin derivatives, cyanide derivatives, malvidin derivatives, petunidin derivatives, and only one derivative of pelargonidin.

The catechins identified belong to gallocatechins, epicatechins, and procyanidins. In addition, other secondary metabolites belonging to the class of phenolic acids and polyphenols were found. These were quantified in all samples, with the except of sinersetin and flavone-(Na^+^), which were found only in ethanol extract samples.

## Conclusion

The *Arbutus unedo* L. plant has been exploited differently over the years in all its parts, especially in traditional medicine. In the food sector, on the other hand, the fruits of *A. unedo* are used to produce liqueurs, wines, jams, and jellies and are rarely consumed as fresh fruit. By analyzing the literature, we have seen that there are few studies on the beneficial effects on human health deriving from the chemical–physical properties of the compounds contained in *A. unedo* fruits: this considers that these studies show an important content of polyphenolic compounds (47, 48). In this study, through the combination of different extraction techniques and different solvents, the phytochemical composition of the strawberry tree fruit in various ripening stages was analyzed. The fruits of *A. unedo* showed an important TPC and a significant scavenger activity on the radical DPPH**^⋅^**. The extraction techniques used, such as ME and UAE, have shown that ultrasound-assisted extraction is more effective in terms of yield and extraction times, against polyphenolic compounds and with scavenger activity on the DPPH**^⋅^** radical. Furthermore, the use of different solvents, pure or mixed, allows to extract specific bioactive components selectively and peculiarly. The use of liquid–liquid separation techniques has also allowed us to purify and concentrate as much as possible the components of our interest for this study. The data obtained from the analyses showed how the TPC varies according to the degree of ripeness going from Red > Orange > Yellow > Green whereas the results deriving from the scavenger activity on the DPPH**^⋅^** root showed how this activity varies according to the degree of ripeness but in a different way by going from Yellow > Red > Orange > Green. The characterization analysis of the chemical components presents in the fractions of selected fruit extracts carried out by LC-MS/MS characterized 56 different compounds of which 19 components were found for the first time in the fruit extracts of *A. unedo*. The chemical composition of the fruit was rich in anthocyanins, catechins, phenolic acids, and polyphenols, in general.

This study showed that the scavenger activity on the DPPH**^⋅^** radical is partly related to the amount of TPC. This study has shown that the qualitative and quantitative chemical diversity detected by LC-MS/MS, relative to the bioactive compounds present in the extracts of *A. unedo*, gives rise, following multivariate analyses, to a discrimination of the studied samples. In general, our results are in good agreement with the results of other papers, although we have found exceptions such as the content of compounds with scavenger activity on the DPPH radical. It is important to underline that we have shown the presence and the trend that many characterized bioactive compounds have depending on the state of maturation. The next step could be the optimization of extraction techniques to trough statistical and mathematical techniques (such as RSM). In addition, a metabolomic and proteomic study linking maturation to the presence of the characterized bioactive compounds could be performed. Given the interesting results obtained, further studies will be necessary to better characterize these compounds to evaluate the bioavailability of the compounds that promote positive effects on health and the consequent use of *A. unedo* extracts to be used in the development of beneficial products such as functional foods with added value, and medicinal supplements.

## Data Availability Statement

The original contributions presented in this study are included in the article/Supplementary Material, further inquiries can be directed to the corresponding author/s.

## Author Contributions

PS, CG, and RSr: conceptualization and project administration. PS and CG: methodology. DZ, APr, and APo: software. RSr, CG, AA, RSh, AG, MT, RG, and PS: validation. AA, GP, AI, and PS: formal analysis and investigation. PS, CG, and RSr: data curation. PS, MT, CG, and RSr: writing—original draft preparation. CG, RSr, DZ, and MT: writing—review and editing. CG and RSr: supervision. All authors contributed to the article and approved the submitted version.

## Conflict of Interest

The authors declare that the research was conducted in the absence of any commercial or financial relationships that could be construed as a potential conflict of interest.

## Publisher’s Note

All claims expressed in this article are solely those of the authors and do not necessarily represent those of their affiliated organizations, or those of the publisher, the editors and the reviewers. Any product that may be evaluated in this article, or claim that may be made by its manufacturer, is not guaranteed or endorsed by the publisher.
