# Supplementary material for: An Endemic Plant of the Mediterranean Area: Phytochemical Characterization of Strawberry Tree (Arbutus unedo L.) Fruits Extracts at Different Ripening Stages
Source: Front Nutr. 2022 Jun 17;9:915994. doi: 10.3389/fnut.2022.915994 (PMC9249387; doi:10.3389/fnut.2022.915994)
Supplement: Supplementary file 1 [file Table_1.docx]

| Table S1. Essential amino acids identified in *A. unedo* fruit extracts by LC-MS analysis (expressed in ng·mL^-1^) | | | | | | | | | | | | |
| --- | --- | --- | --- | --- | --- | --- | --- | --- | --- | --- | --- | --- |
| Amino acid | **VH1** | **GH1** | **AH1** | **RH1** | **VE7** | **GE7** | **AE7** | **RE7** | **VE3** | **GE3** | **AE3** | **RE3** |
| Alanine | 2193.0 | 2114.9 | 2023.4 | 1679.7 | 1711.0 | n.q. | n.q. | n.q. | 1746.8 | 1031.3 | 1946.9 | 1779.7 |
| Valine | 1206.3 | 1187.8 | 775.8 | 711.8 | 763.3 | 86.8 | 123.5 | 94.6 | 6782.1 | 3751.2 | 915.4 | 933.8 |
| Leucine | 321.8 | 150.7 | 35.1 | 36.9 | 302.7 | 139.2 | 48.3 | 79.5 | 467.5 | 265.4 | 50.1 | 99.9 |
| Isoleucine | 5681.6 | 3010.2 | n.q. | n.q. | 8768.5 | 93.2 | 62.1 | 151.8 | 9734.3 | 5498.4 | 2780.8 | 2499.2 |
| Tryptofano | 19331.7 | 725.1 | 763.9 | 1279.3 | 95441.0 | 67.1 | 75.7 | 136.7 | 1088.9 | 604.5 | 1616.6 | 705.7 |
| Tyrosine | 2200.8 | 3682.2 | 2568.3 | 4945.0 | 6469.3 | 294.3 | 134.1 | 264.6 | 3664.7 | 2233.8 | 2437.7 | 3684.6 |
| Phenylalanine | 119.4 | 47.7 | 36.2 | 68.3 | 176.7 | 86.8 | 107.9 | 175.3 | 75.2 | 57.2 | 141.2 | 103.1 |
| Aspartic acid | 4902.2 | 4854.2 | 5016.2 | 4968.0 | 4408.3 | 207.4 | 71.0 | 49.5 | 9250.6 | 5724.4 | 4940.9 | 4564.8 |
| Asparagine | 8140.5 | 6853.3 | 7380.5 | 10918.6 | 11533.8 | 192.0 | 65.2 | 51.1 | 5773.8 | 3733.4 | 4714.4 | 5849.9 |
| Glutamine | 272.0 | n.q. | n.q. | n.q. | 96.7 | 44.1 | 43.0 | 17.6 | 0.5 | n.q. | n.q. | n.q. |
| Glutamic acid | 1556.4 | 1397.1 | 1133.2 | 1148.0 | 1030.9 | 254.1 | 177.2 | 179.9 | 2007.2 | 1307.4 | 932.1 | 1073.8 |
| Arginine | 204.5 | n.q. | 24.4 | 35.2 | 53.3 | 35.2 | 3.4 | 7.8 | 23.0 | 16.9 | 33.9 | 39.4 |
| Lysine | 494.2 | n.q. | n.q. | n.q. | 147.8 | 47.8 | 42.5 | 33.6 | 0.5 | n.q. | 57.6 | n.q. |
| Histidine | 149.9 | 41.7 | 102.1 | 84.5 | 85.5 | 59.6 | 16.0 | 14.8 | 41.9 | 29.8 | 86.6 | 85.8 |
| Serine | 788.6 | 1330.9 | 1103.4 | 868.9 | 821.4 | 69.7 | 18.3 | 29.8 | 2297.6 | 1397.3 | 1118.0 | 1007.2 |
| Threnonine | n.q. | n.q. | n.q. | n.q. | n.q. | 759.0 | 444.2 | 408.7 | 0.5 | n.q. | n.q. | n.q. |
| Cysteine | n.q. | n.q. | n.q. | n.q. | n.q. | n.q. | n.q. | n.q. | 0.5 | n.q. | n.q. | n.q. |
| Cystine | n.q. | n.q. | 63.2 | 53.7 | n.q. | 0.8 | 1.3 | n.q. | 0.5 | n.q. | n.q. | n.q. |
| Methionine | n.q. | 475.6 | 299.9 | 581.0 | 206.8 | 38.7 | 23.0 | 23.1 | 584.6 | 348.8 | 442.8 | 512.5 |
| n.q. = not quantifiable. | | | | | | | | | | | | |
